# BarA-UvrY Two-Component System Regulates Virulence of Uropathogenic *E. coli* CFT073

**DOI:** 10.1371/journal.pone.0031348

**Published:** 2012-02-21

**Authors:** Senthilkumar Palaniyandi, Arindam Mitra, Christopher D. Herren, C. Virginia Lockatell, David E. Johnson, Xiaoping Zhu, Suman Mukhopadhyay

**Affiliations:** 1 Virginia-Maryland Regional College of Veterinary Medicine, University of Maryland, College Park, Maryland, United States of America; 2 Maryland Pathogen Research Institute, University of Maryland, College Park, Maryland, United States of America; 3 Center for Infectious Diseases and Vaccinology, The Biodesign Institute, Arizona State University, Tempe, Arizona, United States of America; 4 Division of Infectious Diseases, Department of Medicine, University of Maryland School of Medicine, Baltimore, Maryland, United States of America; 5 Department of Veterans Affairs, Baltimore, Maryland, United States of America; University of Hyderabad, India

## Abstract

Uropathogenic *Escherichia coli* (UPEC), a member of extraintestinal pathogenic *E. coli*, cause ∼80% of community-acquired urinary tract infections (UTI) in humans. UPEC initiates its colonization in epithelial cells lining the urinary tract with a complicated life cycle, replicating and persisting in intracellular and extracellular niches. Consequently, UPEC causes cystitis and more severe form of pyelonephritis. To further understand the virulence characteristics of UPEC, we investigated the roles of BarA-UvrY two-component system (TCS) in regulating UPEC virulence. Our results showed that mutation of BarA-UvrY TCS significantly decreased the virulence of UPEC CFT073, as assessed by mouse urinary tract infection, chicken embryo killing assay, and cytotoxicity assay on human kidney and uroepithelial cell lines. Furthermore, mutation of either *barA* or *uvrY* gene reduced the production of hemolysin, lipopolysaccharide (LPS), proinflammatory cytokines (TNF-α and IL-6) and chemokine (IL-8). The virulence phenotype was restored similar to that of wild-type by complementation of either *barA* or *uvrY* gene *in trans*. In addition, we discussed a possible link between the BarA-UvrY TCS and CsrA in positively and negatively controlling virulence in UPEC. Overall, this study provides the evidences for BarA-UvrY TCS regulates the virulence of UPEC CFT073 and may point to mechanisms by which virulence regulations are observed in different ways may control the long-term survival of UPEC in the urinary tract.

## Introduction

In humans and animals, pathogenic *E. coli* causes both intestinal and extraintestinal infections [1]. Extraintestinal pathogenic *E. coli* (ExPEC), which includes uropathogenic *E. coli* (UPEC) and avian pathogenic *E. coli*, causes extraintestinal infections in different hosts [2]. Of these, urinary tract infection (UTI) is considered to be the most common bacterial infection in humans [3]. In healthy individuals up to 90% of uncomplicated UTI is caused by UPEC [4]. Recent studies have proposed that prophylactic treatment is unsafe because it may cause antibiotic resistance [5]. UTI is most often caused by ascending bacterial infection contaminating the periurethral area from the lower intestinal tract, then colonizing the bladder via the urethra causing cystitis and in severe cases, further infecting the kidneys via the ureters resulting in pyelonephritis [6]. A hallmark of UPEC infection which is distinct from intestinal pathogenic *E. coli* is that UPEC has to invade the urinary tract for establishing infection. Additionally, UPEC isolates possess genes coding for various virulence factors like adhesins (eg. type 1, P fimbriae), iron acquisition system (eg. aerobactin, enterobactin), host immune evasion mechanisms (eg. capsule) and toxins (eg. cytotoxic necrotizing factor 1, hemolysin) [7], [8].

UPEC's ability to cause common and recurrent infections strongly indicates the presence of virulence factors that facilitate long-term residence or survival and inhabitation in the urinary tract. A variety of virulence genes have been identified in association with *E. coli* mediated urinary tract infections [9]. Several two-component regulatory systems have been involved in the regulation of virulence. The two-component system (TCS) is a major signaling pathway in bacteria that involve phosphotransfering [10]. TCS is widely present in bacteria and regulates gene expressions or protein functions by responding to various environmental signals or stimulations. BarA protein functions as a conserved membrane-associated sensor kinase protein [11]–[13]. The cognate response regulator for BarA is UvrY in *E. coli* [14]. The orthologs of BarA-UvrY TCS in other gram negative γ-proteobacterial species are BarA-SirA in *Salmonella enterica* [15], [16], VarS-VarA in *Vibrio cholerae* [17], GacS-GacA in *Pseudomonas* species [18], [19], respectively. *E. coli* utilizes TCS to respond to the drastic changes in the extracellular environment. For example, *barA* mutants showed sensitivity to oxidative stress due to impairment in catalase expression [20], [21]. Environmental magnesium concentration was a potent stimulus for CsrR-CsrS TCS in group A *Streptococcus* [22]; intestinal short-chain fatty acids and bile alters gene expression and virulence mediated by the BarA-SirA TCS in *Salmonella* [23], [24]. In *E. coli*, BarA-UvrY TCS also regulates the expression of non-coding regulatory CsrB and CsrC RNA, which in turn controls the activity of CsrA protein [16], [25]–[28]. The CsrB and CsrC RNA bind to CsrA protein and prevent it from binding to its target mRNA. CsrA has been elegantly shown to regulate carbon metabolism, flagellar biosynthesis, and biofilm formation [16], [26]–[28].

Previous studies have shown that BarA-UvrY TCS regulates the pathogenicity of avian pathogenic *E. coli* serotype O78∶K80∶H9 by *in vivo* and *in vitro* experiments [29]. The disruptions of the BarA-SirA in *Salmonella enterica* [16], VarS-VarA in *Vibrio cholerae* [17], and GacS-GacA in *Pseudomonas* species [19] lead to remarkable reduction in their virulence. However, the intimate association of virulence and BarA-UvrY TCS in UPEC remains elusive. Therefore, the aim of this study was to investigate the role of the *barA-uvrY* genes in regulating virulence of UPEC CFT073. In addition, we also discussed the potential role of *csrA* gene in regulating the virulence of UPEC CFT073. Revealing of BarA-UvrY TCS involved in regulating UPEC virulence will further allow for a more detailed understanding of uropathogenesis of *E. coli*.

## Materials and Methods

### Bacterial strains, plasmids, cells, and animals

Precise in-frame deletions of *barA*, *uvrY* and *csrA* genes in human uropathogenic CFT073 strain were constructed by using λ Red recombination as described previously [30] using the primers listed in Table 1. The bacterial strains, mutant strains and plasmids for complementation of mutations are listed in Table 2. Primers for amplification of *uvrY* knockout with chloramphenicol cassette, amplification and cloning primers for *barA* and *uvrY* have been described previously [29]. The human uroepithelial SV-HUC-1 and human kidney HK-2 epithelial cell lines were obtained from the American Type Culture Collection (ATCC, Manassas, Virginia). The SV-HUC-1 cell line was grown in complete growth medium F-12K (Invitrogen Life Technologies, Carlsbad, CA) containing 10% fetal bovine serum (Invitrogen). The HK-2 cells were cultured in Keratinocyte serum free medium containing 0.05 mg/ml of bovine pituitary extract and 5 ng/ml of human recombinant epidermal growth factor (Invitrogen). Six to eight week old CBA/J mouse were purchased from National Cancer Institute (Frederick, MD).

**Table 1:** Primers used in this study.

*barA* knockout with chloramphenicol cassette	
OSM 41	5′-CATCGTCGCCATTCCGATATTGTTCGCGCGATTTCG CATATGAATATCCTCCTTAGT-3′
OSM 42	5′-CGACATTATCCATCTCGTCCAACAGTTCCAGCAGCTGTGTAGGCTGGAGCTGCTTC-3′
*csrA* knockout with chloramphenicol cassette	
OSM 39	5′-GAGACCCGACTCTTTTAATCTTTCAAGGAGCAAAGA GTGTAGGCTGGAGCTGCTTC-3′
OSM 40	5′-GAGAAATTTTGAGGGTGCGTCTCACCGATAAAGATGAGACGCGGAAAGACATATGAATATCCTCCTTAGT-3′
TNFα amplification primers	
Forward	5′-AGGCAGTCAGATCATCTTCTCG-3′
Reverse	5′-CCTTGAAGAGGACCTGGGAGTA-3′
IL6 amplification primers	
Forward	5′-TTCGGTCCAGTTGCCTTCTC-3′
Reverse	5′-GTTTTCTGCCAGTGCCTCTTT-3′
IL8 amplification primers	
Forward	5′-CTCTTGGCAGCCTTCCTGA-3′
Reverse	5′-CCTCTGCACCCAGTTTTCCT-3′
GAPDH amplification primers	
Forward	5′-TGGTCTCCTCTGACTTCAACAG-3′
Reverse	5′-AGGAGGGGAGATTCAGTGTG-3′

**Table 2:** *E. coli* strains and plasmids used in this study.

Bacterial Strain or Plasmid	Relevant Genotype	Reference or Source
DH5αK12	*luxS*supE44 Δ(Ф80 Δl*acZM15*) *hsdR17 recA1 endA1 gyrA96 thi-1 relA1*	Invitrogen
CFT073	Wild type	44
SM3009	CFT073 *barA::cm*	this work
SM3010	CFT073 *uvrY::cm*	this work
SM3011	CFT073 *csrA::cm*	this work
SM3012	SM3009 carrying pSM1; Amp^r^	this work
SM3013	SM3010 carrying pSM2; Amp^r^	this work
SM3014	SM3011 carrying pSM7; Amp^r^	this work
CFT073	CFT073 *hlyD::kan*	44
CFT073	CFT073 *hlyD::kan* carrying pSF4000 containing *hlyD*; Cm^r^	44
Plasmids		
pBR322	Cloning vector; Amp^r^	Invitrogen
pSM1	*barA* with EcoRI- EcoRV site of pBR322; Amp^r^	29
pSM2	*uvrY* within the EcoRV-BamHI site of pBR322; Amp^r^	29
pSM7	pCA114, *csrA* under P***_araBAD_***control on pBAD18; Amp^r^, subcloned into pBR322	54

### Ethics Statement

All research protocols involving animals were approved by the Institutional Animal Care and Use Committee of University of Maryland School of Medicine -Baltimore. The experiments were carried out as recommended by the Guide for the Care and Use of Laboratory animals and the animal protocol number is 0106005.

### Ascending urinary tract infection in mouse model

Virulence of the mutants were determined by comparing the urinary tract colonizing abilities of uropathogenic *E. coli* (UPEC) CFT073 [wild-type (WT)], and its isogenic *barA* or *uvrY* mutant in CBA/J mouse by establishing an ascending urinary tract infection (UTI) as previously described [31]. Briefly, groups of 10 female CBA/J mice were co-infected transurethrally either with mixture of 5×10^7^ colony forming unit (CFU) of CFT073 WT and 5×10^7^ CFU of *barA* mutant or with mixture of 5×10^7^ CFU of CFT073 WT and 5×10^7^ CFU of *uvrY* mutant. After 72 hr post-infection, bacterial load was assessed from urine, bladder and kidneys by plating onto LB plates with appropriate antibiotic.

### Chicken embryo lethality assay

Virulence of the bacterial strains was further determined using chicken embryo lethality assay as described previously [29]. Twelve 12-day-old specific-pathogen-free (SPF) chicken embryonated eggs were inoculated with 5×10^3^ CFU of bacteria suspended in 0.1 ml of phosphate buffered saline (PBS) through the allantoic cavity. The bacterial strains inoculated were *E. coli* K-12 DH5α (control), UPEC CFT073 WT, CFT073 *barA* mutant, CFT073 *uvrY* mutant, CFT073 *csrA* mutant, CFT073 *barA/*p*-barA* carrying pSM1, CFT073 *uvrY/*p*-uvrY* carrying pSM2 and CFT073 *csrA/*p*-csrA* carrying pSM7. The plasmids pSM1, pSM2 and pSM7 are all in pBR322-pACYC origin low copy number plasmid vectors to mimic single copy gene complementation. Bacterial strains were grown under static condition in LB broth with appropriate antibiotics for 48 hr at 37°C. Bacterial cells were washed twice and resuspended in PBS. Bacterial suspensions containing 0.1 ml of 5×10^3^ CFU were inoculated into the allantoic cavity using an 18-gauge needle. Glue was used to reseal the holes and the eggs were incubated at 37°C in egg incubator (NatureForm, FL). At every 12 hr post challenge, eggs were monitored by candling and reported as alive or dead based on the integrity of the venous system and the activity of embryo movement.

### Attachment and invasion assays

Infections of human uroepithelial cell line SV-HUC-1 were performed in ∼70% confluence monolayer by growing cells in 6-well plates at 37°C for 48 hr. Type 1 pilus formation was induced by growing bacteria in LB media for 48 hr under static condition.

P pilus was induced by growing bacteria on tryptic soy agar plates. The experiments were performed under both culture conditions and similar results were observed for attachment and invasion. Adherence assays were performed as described previously [32].

Uroepithelial cells were infected with a 10∶1 multiplicity of infection (MOI). Before infection, fresh medium was used. Tissue culture plates were centrifuged 600× g for 5 minutes and then incubated at 37°C for 2 hr. Subsequently, 20 µl of 5% Triton X-100 was used to lyse cells from three wells and then plated onto LB agar plates to enumerate the bacterial load for both intra- and extracellular bacteria. Adherent bacteria calculated from infected cells were washed with PBS for five times, finally lysed in 1 ml of 0.1% Triton X-100 and plated onto LB agar plates to measure the number of adherent bacteria.

To calculate invasion frequencies, a set of three infected wells were washed with PBS for five times, then bactericidal antibiotic gentamicin (100 µg/ml), which does not penetrate uroepithelial cells, was added to the infected cells to kill adhered extracellular bacteria and incubated for another 4 hr. At the end of incubation the cells were completely washed with PBS, lysed with 1 ml of 0.1% Triton X-100. Bacterial load was calculated by plating onto LB agar plates with appropriate antibiotics. Attachment index was determined as CFU/ml of the adherent bacteria divided by total bacterial inoculum (CFU/ml). Invasion index was determined as number of bacteria surviving gentamicin treatment divided by the total number of bacteria present before gentamicin incubation.

### Cytotoxicity assay on human kidney (HK-2) cells

The HK-2 cells grown in 96-well plate with 70% confluence monolayer were treated with 50 µl of filter-sterilized culture supernatants from equal number of bacterial cells grown in LB media under static condition at 37°C for 48 hr for two passages with appropriate antibiotics. Cytotoxic effects were analyzed by cell proliferation assay (colorimetric tetrazolium WST-1) under the absorbance at 450 nm as recommended by manufacturer (Roche, USA). Pilot experiments were performed to determine the optimum incubation time for cytotoxic effects. After the cells were treated with bacterial supernatants for 180 minutes, 10 µl of tetrazolium salt WST-1 was added to each well and the cells were incubated for an additional 180 minutes at 37°C. Absorbance was read at 450 nm using a PerkinElmer Victor3 plate reader.

### Hemolysin assay

Hemolysin assay with sheep erythrocytes was performed as described [33] with minor modifications. Similar results were obtained when bacterial strains were grown either in LB (data not shown) or in artificial urine medium. Simple artificial urine medium was prepared as described previously [34]. The artificial urine medium was adjusted to pH 6.5 and sterilized with 0.2-µm-pore-size filter. Filter sterilized bacterial culture supernatants harvested at an optical density of 600 nm (OD_600_) of 0.4 was used. 100 µl of 2% sheep erythrocytes (RBC) suspended in PBS was mixed with 200 µl of filter sterilized supernatants and incubated at 30.5°C for 7 hr. The incubation time and temperature were standardized with initial experiments in our assay for showing maximum difference. Unlysed cells were pelleted and absorbance of the supernatant was measured at 405 nm.

### Preparation of secreted protein

Secreted proteins in supernatant were prepared as described previously [35]. Bacterial cells were pelleted by centrifugation at 10,000 rpm for 30 minutes at 4°C and supernatant was filtered using 0.2-µm-pore-size filter. Proteins were precipitated with trichloroacetic acid (25% wt/vol final concentration) on ice for 4 hr, washed with acetone and analyzed in Novex 4–20% Tris-Glycine gel (Invitrogen). The gel was stained with Coomassie blue and appropriate bands were excised and subjected to mass spectrometric analysis (The University of Kansas Medical Center, Kansas City, KS).

### Extraction of lipopolysaccharide (LPS)

LPS extraction was performed by a modified phenol-chloroform method using a LPS extraction kit (Intron Biotechnology, Boca Raton, FL). Bacterial cultures were grown under static condition in tryptic soy broth at 37°C for 48 hr. Bacterial cells were normalized for LPS extraction and bacterial cultures were processed according to the manufacturer instructions. LPS pellet was washed with 1 ml of 70% ethanol and then air dried completely. Finally, LPS was dissolved in 70 µl of double distilled water by boiling for 1 minute. LPS was separated in 12% SDS-PAGE gel under reducing conditions and visualized with silver staining as recommended by manufacturer (FastSilver, G Biosciences, St. Louis, MO). During the fixation, 0.7 g of periodic acid was added to 100 ml of fixing solution to oxidize the carbohydrate as described previously [36]. LPS image was photographed by using Kodak Electrophoresis Documentation and Analysis System (EDAS) 290 camera. The LPS concentration was calculated with densitometry by comparing with a standard from *E. coli* LPS (Sigma, St. Louis, MO).

### Quantitative real time PCR (qRT-PCR)

SV-HUC-1 uroepithelial cells were grown to confluence in 6-well plates. The cells were treated with equal amount of purified LPS and incubated for 16 hr at 37°C or with whole bacterial cells with MOI (10∶1, bacteria: SV-HUC-1 cells) for 4 hr at 37°C. The bacterial cells were grown in LB media under static condition for 48 hr at 37°C with appropriate antibiotics. Total RNA was isolated from the infected SV-HUC-1 cells using the TRIzol reagent (Invitrogen). The extracted RNA was treated with TURBO DNase (Ambion, Austin, TX) and further purified using Qiagen RNeasy mini-columns (Qiagen, Valencia, CA). For RT-PCR, first-strand cDNA was synthesized from 5 µg of total RNA using Superscript II (Invitrogen) with 50 ng of random hexamers (Invitrogen). Internal gene-specific primers were used to amplify TNF-α, IL-6, IL-8 and internal control glyceraldehyde 3-phosphate dehydrogenase (GAPDH). The primer sequences to amplify these genes were listed in Table 1. For qRT-PCR, 10 ng of first-strand cDNA was amplified separately with 10 µM each of gene or GAPDH-specific primers in a 25-µl total volume of SYBR green 1 PCR master mix using a PTC-200 Opticon Cycler (Biorad, Hercules, CA). The ΔC*_T_* values between samples were normalized to GAPDH product and calculated as ΔC*_T_* = [C*_T_* of uninfected Cytokine (eg. Mock TNF-α)−C*_T_* of infected Cytokine (eg. Infected TNF-α)]−[C*_T_* of uninfected GAPDH (mock)−C*_T_* of infected GAPDH]. Because sample was duplicated by each PCR amplification cycle, the fold difference in the initial concentration of each transcript is determined as 2^−ΔΔC*T*^.

## Results and Discussion

### Mutation in *uvrY* or *barA* decreases the virulence in a mouse UTI model

**Figure 1:**
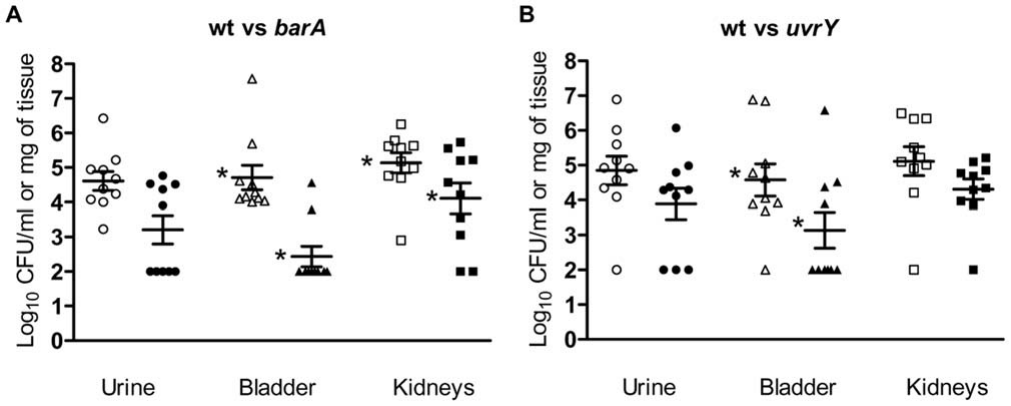
Mutation in *barA or uvrY* reduces virulence in murine ascending UTI. CBA/J mice were infected transurethrally with mixture of 5×10^7^ CFU of WT bacteria and 5×10^7^ CFU of mutant bacteria. **A**. Bacteria were recovered 72 hr later from mice infected with CFT073 WT urine (○), SM3009 (*barA::cm*) urine (•), CFT073 WT bladder (Δ), SM3009 (*barA::cm*) bladder (▴), CFT073 WT kidneys (□), SM3009 (*barA::cm*) kidneys (▪) and were expressed in Log_10_ CFU. **B**. Bacteria were recovered 72 hr later from mice infected with CFT073 WT urine (○), SM3010 (*uvrY::cm*) urine (•), CFT073 WT bladder (Δ), SM3010 (*uvrY::cm*) bladder (▴), CFT073 WT kidneys (□), SM3010 (*uvrY::cm*) kidneys (▪) and were expressed in Log_10_ CFU. The results were representative of two independent experiments. Star denotes P<0.01.

The UPEC can cause cystitis or pyelonephritis. The UPEC originate from distal gut, colonize the vagina and/or ascend the urinary tract to the bladder via the urethra [37], [38]. The UPEC have the ability to invade the urinary tract and develop biofilms [39]. To investigate the effect of the *uvrY or barA* mutant on bacterial pathogenesis, we developed ascending urinary tract infection via transurethral catheterization in a mouse model. To evaluate the virulence, bacterial load was determined in various tissues and urine. Mutation in either *barA* or *uvrY* exhibited reduced colonization as compared to the wild-type in mouse ascending UTI model (Figure 1). The *barA* mutant bacteria colonized less efficiently in both bladder and kidneys, while *uvrY* mutant bacteria colonized less efficiently in the bladder of the mice when compared to the wild-type. The bacterial loads of *barA* mutant colonized in bladder and kidneys were significantly reduced by two and one log10 CFU/gram of tissue as compared to wild-type (*P<0.01). The *uvrY* mutant demonstrated significant reduction of bacterial loads in bladder by one log_10_ CFU/gram of tissue when compared to that of wild-type (*P<0.01, Figure 1). Our result showed that the load of mutant was slightly reduced in the urine by approximately one log_10_ CFU/ml, which may further explain the poor colonization of the bladder and kidneys. Using *E. coli* DS17 it has been shown that the *uvrY* mutant has a lesser fitness for survival in urine in a primate infection model [40]. Here, we have showed the extent of colonization of both *barA* and *uvrY* mutant in the tissues of the urogenital system.

### Mutation in *uvrY* or *barA* reduces virulence in a chicken embryo lethality assay

**Figure 2:**
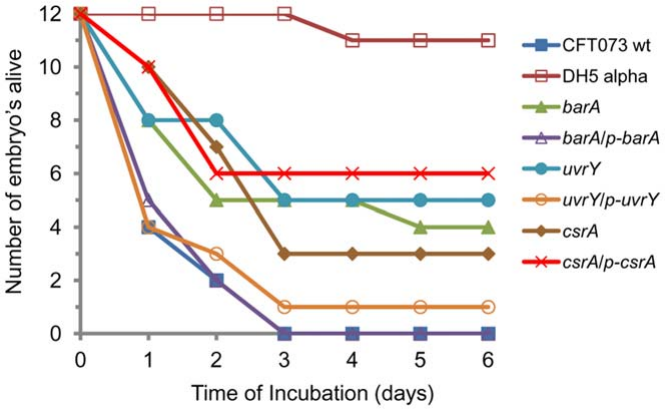
Chicken embryo infections by UPEC CFT073 and its mutant. A set of twelve 12-day-old SPF embryonated eggs were inoculated through allantoic cavity with 5×10^3^ CFU of bacteria CFT073 WT (▪), DH5αΚ12 (□), SM3009 (*barA::cm*) (▴), SM3012 (*barA/*p*-barA*) carrying pSM1 (Δ), SM3010 (*uvrY::cm*) (•), SM3013 (*uvrY*/p-*uvrY*) carrying pSM2 (○), SM3011 (*csrA::cm*) (♦) and SM3014 (*csrA*/p-*csrA*) carrying pSM7 (X). The results were scored as live, morbid or dead. The results were representative of two independent experiments.

The virulence of BarA-UvrY TCS of avian pathogenic *E. coli* strain χ7122 was shown using chicken embryo lethality assay [29]. In this assay, the virulence can be determined by the number of embryos killed within a given period of time. In our study, all the embryos infected with WT strain were dead (100%) 72 hr post inoculation, whereas the mortality of the embryos inoculated with *barA* or *uvrY* mutant decreased to 67% (4 out of 12 survived) and 58% (5 out of 12 survived) even up to 6 days post inoculation, respectively. The reduction in virulence is significant (P<0.01) (Figure 2). The reduced virulence were reestablished to that of WT CFT073 when the respective mutant genes were provided *in trans*. Complementation was 100% in *barA/*p*-barA* strain and 91% in *uvrY/*p*-uvrY* strain. These results obtained from mutation of either *barA* or *uvrY* were well correlated with that of mouse UTI. Overall, we observed a phenomenon, wherein mutation of *barA* or *uvrY* reduced the virulence and the complementation of these genes *in trans* reversed the phenotype.

### Mutation in *uvrY* reduces the invasion to uroepithelial (SV-HUC-1) cells

The roles of *barA* and *uvrY* in mediating attachment or invasion in cultured uroepithelial cells were investigated by using standard gentamicin protection assay [32]. Mutation in *uvrY* significantly reduced the invasion abilities by two logs (∼100-fold) in cultured ureter (SV-HUC-1) (**p≤0.01, relative to WT) epithelial cells (Table 3). However, the bacterial attachment was unaltered and there was no significant difference between WT and mutant bacteria. Complementation of *uvrY* mutant *in trans* expressing *uvrY* gene restored the invasion abilities in cultured uroepithelial cells similar to the level of wild-type (Table 3). However, deletion of *barA* had no effect on the attachment and invasion in ureter (SV-HUC-1) cells. These results suggest that as a transcriptional regulator, UvrY plays an important role in determining virulence. Mutation in *uvrY* causes greater level of attenuation as compared to mutation in *barA*.

**Table 3:** Mutation in *uvrY* reduces invasion of *E. coli* CFT073 strain to ureter (SV-HUC-1) uroepithelial cells.

Genotype	Initial cells (log10 CFU /ml)	Attached and invaded after 2 h (log10 CFU / ml)	Invaded fraction surviving after 4 h (log 10 CFU / ml)	Calculated attached bacteria (log 10 CFU/ml)	Attachment Index	Invasion Index
CFT073 WT	8.4±0.9	7.4±0.8	3.1±0.5	7.5±0.8	7.7×10^−2^	6.8×10^−5^
*barA*	8.3±0.9	7.2±0.9	3.0±0.5	7.2±0.9	8.3×10^−2^	6.6×10^−5^
*barA/p-barA*	8.9±0.9	7.7±0.9	3.3±0.5	7.7±0.9	6.3×10^−2^	4.7×10^−5^
*uvrY* **	8.6±0.9	6.8±0.8	0.7±0.1	6.8±0.8	1.7×10^−2^	7.1×10^−7^
*uvrY*/p-*uvrY*	8.8±0.9	7.6±0.9	2.9±0.5	7.6±0.9	6.3×10^−2^	1.9×10^−5^
*csrA*	7.7±0.9	6.6±0.8	2.2±0.3	6.6±0.8	8.9×10^−2^	3.6×10^−5^
*csrA*/p-*csrA*	7.7±0.9	6.6±0.8	0.7±0.1	6.6±0.7	7.8×10^−2^	1.3×10^−6^

Results represent mean ± SD from three individual experiments.

**Mutation in *uvrY* significantly reduces the virulence (p≤0.01, relative to WT).

### Mutation in *barA* or *uvrY* reduces the cytotoxic effect to cultured human kidney (HK-2) cells

**Figure 3:**
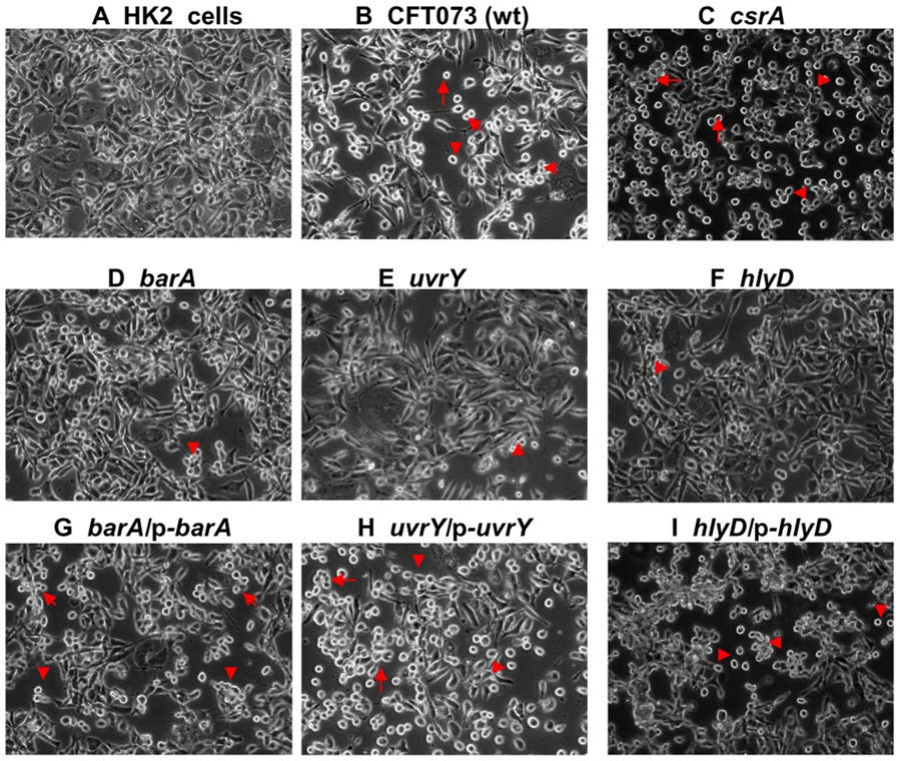
Phase-contrast photomicrographs of human kidney (HK-2) cells. Photomicrographs were taken 6 hr later from either untreated control (HK-2 cells) (A), or treated with supernatants from CFT073 WT (B), *csrA* (C), *barA* (D), *uvrY* (E), *hlyD* (F), *barA*/p-*barA* (G), *uvrY*/p-*uvrY* (H) and *hlyD*/p*-hlyD* (I). The rounding and detachment of cells in the treated monolayer are marked with arrow or arrowhead. The pictures were representative of three individual experiments.

Mutation in either *barA* or *uvrY* reduced the cytotoxic effects of the bacterial supernatants on human kidney HK-2 cells detected by addition of WST-1 reagent to infected cells (Figure 3 & Table 4). Bacterial supernatants significantly inhibited the growth because only 21% of the treated cells were alive in WT (Figure 3B). In contrast, the deletion of *barA* (Figure 3D), *hlyD* (Figure 3F) and *uvrY* (Figure 3E) significantly attenuated the cytotoxicity with 38% (*, p≤0.01), 35% (*, p≤0.01), and 68% (**, p≤0.001) of the cells alive, respectively, when compared to the cells survived from wild-type (Table 4). Complementation of the mutants *in trans* restored the virulence to the similar level of wild-type. These results also demonstrated the cytotoxicity of *uvrY* mutant is significantly reduced compared to the *barA* mutant (*** p≤0.0001). Taken together, our results suggest that soluble factors, like cytotoxic proteins or LPS, in bacterial culture supernatants may contribute to the virulence of the bacteria.

**Table 4:** Mutation in *barA* or *uvrY* reduces and *csrA* increases the cytotoxic effect to human kidney (HK-2) cells.

Genotype	Percentage of survival^a^ (relative to control)
Control	100
CFT073 WT	21±3
*hlyD*	35±2*
*hlyD*/p-*hlyD*	21±2
*barA*	38±3*
*barA/*p*-barA*	22±2
*uvrY*	68±2**^,^***
*uvrY/*p-*uvrY*	22±3
*csrA*	13±4
*csrA/*p*-csrA*	36±3

a Data obtained from three independent experiments with 6 replicates per condition.

(*, p≤0.01; **, p≤0.001, relative to WT, *** p≤0.0001, relative to *barA* mutant).

### Mutation in *barA* or *uvrY* affects hemolysin secretion

**Figure 4:**
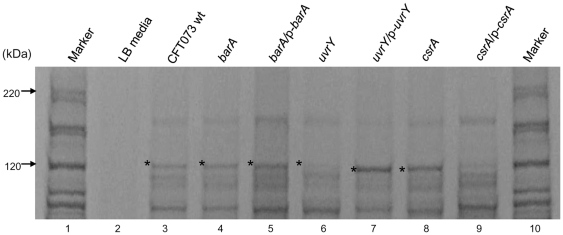
Protein profiles of wild type and mutant bacteria supernatant. Secreted supernatants were filter purified and separated on 4–20% SDS-PAGE gel and stained with Coomassie blue. Bands of the interest were incised and subjected to mass spectrometry analysis. Star indicates the location of the hemolysin protein band. The results were representative of three individual experiments. Lanes: 1. protein marker, 2. LB media, 3. CFT073 wt, 4. *barA*, 5. *barA/*p*-barA*, 6. *uvrY*, 7. *uvrY*/p-*uvrY*, 8. *csrA*, 9. *csrA*/p*-csrA*, 10. protein marker.

Differential expression of virulence factors in UPEC might be an important factor for colonizing either in the bladder causing cystitis or in kidney resulting in pyelonephritis. Since the *barA* or *uvrY* mutant showed decreased colonization in mouse UTI model, chicken embryo killing and reduced cytotoxicity to cultured HK-2 cells, we further compared the secreted protein profiles in the culture supernatants from the various mutants and wild-type. By Coomassie blue staining, we identified a unique band of approximately 110 kDa that was reduced in its expression level in either *barA* or *uvrY* mutant. By mass spectrometric analysis, this band was further identified as Hemolysin (HlyA) [41], [42] (Figure 4). The exotoxin HlyA is one of the virulence factors associated with pathogenesis; the production of hemolysin protein contributes to the virulence of extra-intestinal pathogenic *E. coli* infections [33], [43], [44]. *E. coli* bacterial suspensions containing hemolysin treated onto cultured human kidney proximal tubular epithelial cells resulted in highly elevated cytotoxicity; transurethral challenge in CBA mice resulted in pyelonephritis [44]. The HlyA was down-regulated in the *uvrY* mutant (Figure 4, lane 6) compared to wild-type (Figure 4, lane 3). Furthermore, we investigated the hemolytic function of the secreted hemolysin protein from bacterial culture supernatant by using sheep erythrocytes at 30.5°C for 7 hr. The hemolytic activity of *barA* and *uvrY* mutants was significantly reduced when compared to wild-type (***, p≤0.001). There was more than 5-fold or 7-fold decrease in hemolytic activity in the mutant *barA* or mutant *uvrY* respectively (Table 5). Functional complementation of both mutant genes by plasmids restored the hemolytic activity similar to the level of wild-type (Table 5). The decreased activity of the exotoxin HlyA may also contribute to the reduction of the virulence in *barA* or *uvrY* mutant bacteria. Taken together, the hemolytic activities in supernatants from the *barA* or *uvrY* mutants might contribute and well correlated to the reduced cytotoxic effects against human kidney HK-2 cells and decreased chicken embryo mortality.

**Table 5:** Mutation in *barA* or *uvrY* decrease and *csrA* increase hemolysis of sheep erythrocytes.

Genotype	Hemolysis of sheep erythrocytes (OD_405_)^a^
CFT073 WT	2.7±0.19
*hlyD*	0.57±0.09
*hlyD*/p-*hlyD*	10.3±0.28
*barA*	0.53±0.12***
*barA/*p*-barA*	1.85±0.10
*uvrY*	0.35±0.14***
*uvrY/*p-*uvrY*	3.6±0.18
*csrA*	3.9±0.28**
*csrA/*p*-csrA*	0.39±0.22***

a Data obtained from mean values ± standard deviations from three different experiments.

(**, p≤0.01; ***, p≤0.001 compared to WT).

### Deletion of *uvrY* affects LPS profile

**Figure 5:**
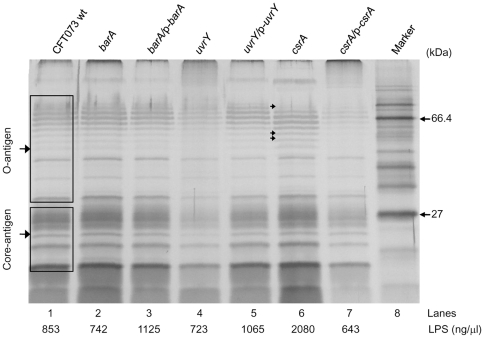
Lipopolysaccharide profiles of wild type and mutant bacteria. LPS was extracted from equal number of cells and separated on 12% SDS-PAGE gel and visualized with silver stain. The upper bands represent the O antigen and the lower bands represent the core LPS antigen. The results were representative of three individual experiments. Lanes: 1. CFT073 wt, 2. *barA*, 3. *barA*/p*-barA*, 4. *uvrY*, 5. *uvrY*/p-*uvrY*, 6. *csrA*, 7. *csrA*/p*-csrA*, 8. protein marker.

is a potent endotoxin responsible for gram negative septicemia [45] and also responsible for the production of a variety of proinflammatory cytokines followed by septic shock and disseminated intravascular coagulation of the infected animals [46]. Next, we investigated the role of various mutants in UPEC lipopolysaccharide biosynthesis. As shown in the Figure 5, LPS was isolated from equal number of cells and resolved in reducing 12% SDS-PAGE. The LPS profile of the *uvrY* mutant exhibited visible differences in the core and O-antigen compared to that of LPS from wild-type. Mutation in *uvrY* (Figure 5, lane 4), caused differences in both migration pattern of O-antigen bands and quantity of the LPS as compared to the LPS from wild-type (Figure 5, lane 1). Some bands of the O-antigen were missing and few other bands were less prominent when compared LPS in WT CFT073. Complementation of the *uvrY* mutant (Figure 5, lane 5) *in trans* restored the WT phenotype of LPS. In *csrA* mutant LPS, one high molecular weight O-antigen band became less prominent and few low molecular weight O-antigen bands showed more prominence (indicated by arrows in Figure 5, lane 6). The *csrA/*p*-csrA* complemented strain had reduced LPS expression (Figure 5, lane 7). Quantification of the LPS from equal number of *E. coli* cells (4×10^9^) by densitometry, titrated the amount of LPS from *uvrY* (Figure 5, lane 4, 723 ng/µl) mutant strain was partially reduced when compared to the wild-type (Figure 5, lane 1, 853 ng/µl) and the complementation of *uvrY* mutant restored the WT phenotype (Figure 5, lane 5, 1065 ng/µl). One possible mechanism is that mutation of *uvrY* may modulate the *rfa* gene cluster responsible for LPS biosynthesis resulting in differential expression of LPS. Loss of LPS or changes in LPS profile may also lead to reduction in colonization and invasiveness. Also rough strains do not persist in vivo as efficiently as the smooth strains. This might help in explaining *uvrY* mutant is not so invasive or have low level of colonization in mouse UTI model.

### Mutation in *uvrY* down-regulates inflammatory cytokines

LPS is an integral part of outer membrane of UPEC. Either bacterial infection or LPS treatment triggers cytokine production in mucosal tissue [47]–[49]. The epithelial cells lining urinary tract play a major role in host pathogen interaction by secreting various cytokines in response to infections. First, UPEC invades and colonizes in uroepithelial cells, which triggers the release of proinflammatory cytokines. It has been shown that the uroepithelial cell lines secrete interleukin-6 (IL-6) and chemokine IL-8 when stimulated by UPEC [47], [49]. During gram negative septicemia, the proinflammatory cytokines like tumor necrosis factor α (TNF-α) and IL-6 and chemokine IL-8 activate the inflammatory cascade and trigger the systemic infection [48]–[51]. Here, we further investigated the cytokine responses of human uroepithelial cell line SV-HUC-1 cells to infection with either whole bacterial cells or treatment with purified LPS alone by quantitative RT-PCR analysis (Table 6). Both whole bacterial cells and LPS treatment stimulated the mRNA expressions of TNF-α, ΙL-6 and IL-8. Their expression levels were higher in SV-HUC-1 cells infected with whole bacterial cells than in cells treated with LPS alone. Possible explanation for this higher expression of TNF-α, ΙL-6 and IL-8 from stimulation with whole bacterial cells may be due to surface expression of virulence factors like Type 1 or P fimbriae, capsule, outer membrane proteins or O-specific antigen and toxins including hemolysin and LPS. The infection with WT bacteria and its LPS treatment had the highest expression of all three cytokines. These results support the previous findings [47]–[49] while infection with *uvrY* mutant bacteria down-regulated TNF-α, IL-6 and IL-8 by ∼4, 5 and 9-folds compared to the WT bacteria (**, p≤0.01) (Table 6). The cells treated with purified LPS from *uvrY* mutant also down-regulates the cytokines TNF-α and IL-6 by ∼3-folds compared to the WT LPS (*, p≤0.05). The *uvrY*/p-*uvrY* complemented strain restored the effects on the level of cytokine production similar to that of wild-type (Table 6). The difference in the LPS profile pattern (Figure 5) may contribute to the lowered expressions of these cytokines. These factors might also contribute to the reduction in virulence of the *uvrY* mutant.

**Table 6:** Mutation in *uvrY* gene down regulates inflammatory cytokine expression.

Genotype	Fold change in mRNA levels of SV-HUC-1 ureter cells treated with					
	Purified LPS (10 ng)			Whole bacterial cells		
	TNFα	IL6	IL8	TNFα	IL6	IL8
CFT073 WT	3.7±0.3↑	5.5±0.4↑	2.2±0.3↑	8.7±0.6↑	17.4±0.9↑	12.08±0.5↑
*uvrY*	1.0±0.6↓*	2.0±0.1↓*	1.6±0.4↓*	2.1±0.3↑**	3.6±0.1↑**	1.4±0.4↓**
*uvrY/*p*-uvrY*	2.9±0.3↑	4.9±0.3↑	2.1±0.2↑	8.2±0.9↑	17.8±0.5↑	12.1±0.7↑
*csrA*	1.8±0.2↑*	3.6±0.2↑*	1.5±0.1↑	3.6±0.6↑*	6.1±0.4↑*	4.3±0.4↑**
*csrA/*p-*csrA*	1.1±0.4↑	1.8±0.7↑	1.0±0.2↑	2.0±0.2↑	3.2±0.3↑	1.5±0.7↑

The values are mean ± SD of three independent experiments with triplicate samples.

The downward arrow indicates down regulation compared to the WT.

(*, p≤0.05; **, p≤0.01 compared to WT).

Majority of UTI were caused by UPEC [38], [52]. Our results with murine UTI showed the important roles of BarA-UvrY TCS in the virulence of UPEC. Mutation with *barA* and *uvrY* reduced the invasion and colonization in bladder and kidneys, decreased chicken embryo killing, and reduced cytotoxicity to HK-2 cells (Figure 1, 2& 3; Table 4). There was 1–2 log reduction in bacterial colonization and 58–67% reduction in chicken embryo killing, suggesting that the pathogenicity and virulence factors of UPEC CFT073 were controlled by various virulence determinants. Hence, mutation in BarA-UvrY TCS was less efficient and various other TCS and signaling pathways may also contribute to the virulence of UPEC CFT073. Between the mutations in BarA-UvrY TCS, effect of *uvrY* mutation is more pronounced and less virulent as compared to mutation in *barA*, a membrane sensor protein. This conclusion is strongly supported by reduction of other virulence determinants like LPS, hemolysin and proinflammatory cytokines and chemokine (Figure 4& 5, Table 5 &6).

It has been shown that CsrA is a RNA binding protein. More interestingly, mutation in *csrA* increased the virulence of UPEC CFT073. This conclusion is supported by several evidences. First, deletion of *csrA* in strain CFT073 increased the virulence to 75% (3 out of 12 survived) in a chicken embryo lethality assay (Figure 2). The increased virulence was reduced to 50% in a complemented *csrA/*p*-csrA* strain. This result is in agreement with a previous study that mutation of *csrA* in *E. coli* K-12 enhanced biofilm formation by regulating intracellular glycogen biosynthesis and catabolism and over expression of *csrA* repressed biofilm formation [28]. These results suggest that *csrA* gene represses certain virulence factors; CsrA may exhibit distinct mechanism from BarA-UvrY TCS in modulating virulence factors. Second, deletion of *csrA* gene which controls carbon metabolism and flagellum biosynthesis [26], [27] resulted in unaltered invasiveness in ureter (SV-HUC-1) cells while the *csrA*/p*-csrA* complemented mutant strain reduced the invasiveness by one log (∼10-fold) in SV-HUC-1 uroepithelial cells (Table 3).

The differences exhibited by the invasion ability of *csrA* mutant in ureter (SV-HUC-1) may be due to the growth rate of the *csrA* mutant or owing to the changes in the expression of virulence factor during invasion. Indeed, mutations of *csrA* gene affect the growth of the bacteria and these mutants tend to grow slowly. Third, *csrA* mutant was more cytotoxic (Figure 3C) compared to its wild-type (Figure 3B), where only 13% of treated HK-2 cells (Table 4) were alive and mutation in *csrA* gene resulted in moderate increase in hemolytic activity and its complementation in plasmid decreased the hemolytic activity, indicating that CsrA represses the hemolysin expression (Table 5). Fourth, the *csrA* mutant had the highest concentration of LPS (Figure 5, lane 6, 2080 ng/µl) and *csrA*/p-*csrA* complemented strain led to reduction in the level of LPS expression (Figure 5, lane 7, 643 ng/µl). Interestingly, even though the *csrA* mutant had the highest concentration of LPS (Figure 5, lane 6, 2080 ng/µl) from equal number of bacterial cells, SV-HUC-1 cells treated with equal amount of LPS (10 ng/µl) did not produce the cytokine levels like that of wild-type (Table 6). Taken together, our assay demonstrated that increased virulence determinants, like hemolysin activity and LPS, may contribute to the enhanced virulence in *csrA* mutation and over-expression of *csrA* suppresses the bacterial virulence. However, the increase in virulence seen with the csrA mutant in these *in vitro* experiments needs to be further verified by *in vivo* animal experiments.

In spite of this, our *in vitro* experimental results are in agreement with the previous study that csrA represses *pgaABCD* transcript involved in the synthesis of polysaccharide adhesion, thereby repressing biofilm formation in *E. coli* [53], but different from another study that mutation of *csrA* gene in *Salmonella enterica* serovar Typhimurium reduces the invasion of HEp-2 epithelial cells and expression of *Salmonella* pathogenicity island 1 (SPI1) invasion genes [54]. However, over-expression of *csrA in trans* also suppress the expression of SPI1 invasion genes [54]. Also, in *Legionella pneumophila*, over-expression of CsrA suppress virulence associated traits and mutation of *csrA* gene result in increased virulence associated gene *letE*, stationary-phase sigma factor, RpoS and enhance *flaA* and *fliA* genes resulting in premature flagellation [55]. These studies, including ours, demonstrated that CsrA acts as both repressor and enhancer of invasion genes and its expression could be tightly regulated. The future study can be directed to examine the expression level of CsrA protein in the *uvrY* mutant and establish a functional relationship between BarA-UvrY TCS and CsrA in regulating the expression of virulence genes either positively or negatively.

In summary, our work further delineates the role of BarA-UvrY TCS in regulating various virulence factors. In UPEC, the Cpx two-component signal transduction system, CpxA/CpxR controls P pilus biosynthesis and regulates phase variation of *pap* and other virulence factors [56]. It is also reported that pyelonephritogenic *E. coli* strains are more cytotoxic to cultured human renal tubular epithelial cells and the cytotoxin hemolysin contributes to this potent virulence as well [44]. A previous study showed that disruption of the BarA-UvrY TCS reduced the fitness of the *uvrY* mutant in a monkey cystitis model [40]. Similarly, in our assay, mutation in *uvrY* reduces the cytotoxicity to the cultured human kidney (HK-2 cells) and hemolysin production (Figure 3 & 4, Table 4 & 5). Previously our microarray data showed and we also hypothesize that BarA-UvrY two-component system may regulate the expression of *pap* gene cluster encoding P pilus, fimbrial genes encoding Type 1 fimbriae, outer membrane protein such as *ompC* or LPS biosynthesis genes like *rfa* gene cluster, *hly* locus encoding hemolysin synthesis and secretion, which may directly or indirectly contribute to the virulence of the UPEC [57]. Further investigation is needed to understand the role of these genes in the pathogenesis of UPEC in the UTI. Taken together, our results show that BarA-UvrY TCS regulates various virulence determinants contributing to the pathogenicity of the UPEC CFT073.
